# Crystal Structure, Spectroscopic Characterization, Antioxidant and Cytotoxic Activity of New Mg(II) and Mn(II)/Na(I) Complexes of Isoferulic Acid

**DOI:** 10.3390/ma14123236

**Published:** 2021-06-11

**Authors:** Monika Kalinowska, Ewelina Gołębiewska, Liliana Mazur, Hanna Lewandowska, Marek Pruszyński, Grzegorz Świderski, Marta Wyrwas, Natalia Pawluczuk, Włodzimierz Lewandowski

**Affiliations:** 1 Department of Chemistry, Biology and Biotechnology, Institute of Civil Engineering and Energetics, Faculty of Civil Engineering and Environmental Science, Bialystok University of Technology, Wiejska 45E Street, 15-351 Bialystok, Poland; e.golebiewska@pb.edu.pl (E.G.); g.swiderski@pb.edu.pl (G.Ś.); martawyrwas024@gmail.com (M.W.); natalia.pawluczuk97@gmail.com (N.P.); w-lewando@wp.pl (W.L.); 2 Department of General and Coordination Chemistry and Crystallography, Institute of Chemical Sciences, Faculty of Chemistry, Maria Curie-Skłodowska University, M. C. Skłodowskiej Sq. 2, 20-031 Lublin, Poland; l.mazur@poczta.umcs.lublin.pl; 3 Institute of Nuclear Chemistry and Technology, 16 Dorodna Street, 03-195 Warsaw, Poland; h.lewandowska@ichtj.waw.pl (H.L.); m.pruszynski@ichtj.waw.pl (M.P.); 4 NOMATEN Centre of Excellence, National Centre of Nuclear Research, 7 Andrzeja Soltana Street, 05-400 Otwock, Poland

**Keywords:** plant phenolic compounds, metal complexes, 3-hydroxy-4-methoxycinnamic acid, isoferulic acid, magnesium, manganese, antioxidant activity

## Abstract

The Mg(II) and heterometallic Mn(II)/Na(I) complexes of isoferulic acid (3-hydroxy-4-methoxycinnamic acid, IFA) were synthesized and characterized by infrared spectroscopy FT-IR, FT-Raman, electronic absorption spectroscopy UV/VIS, and single-crystal X-ray diffraction. The reaction of MgCl_2_ with isoferulic acid in the aqueous solutions of NaOH resulted in synthesis of the complex salt of the general formula of [Mg(H_2_O)_6_]⋅(C_10_H_9_O_4_)_2_⋅6H_2_O. The crystal structure of this compound consists of discrete octahedral [Mg(H_2_O)_6_]^2+^ cations, isoferulic acid anions and solvent water molecules. The hydrated metal cations are arranged among the organic layers. The multiple hydrogen-bonding interactions established between the coordinated and lattice water molecules and the functional groups of the ligand stabilize the 3D architecture of the crystal. The use of MnCl_2_ instead of MgCl_2_ led to the formation of the Mn(II)/Na(I) complex of the general formula [Mn_3_Na_2_(C_10_H_7_O_4_)_8_(H_2_O)_8_]. The compound is a 3D coordination polymer composed of centrosymmetric pentanuclear subunits. The antioxidant activity of these compounds was evaluated by assays based on different antioxidant mechanisms of action, i.e., with ^•^OH, DPPH^•^ and ABTS^•+^ radicals as well as CUPRAC (cupric ions reducing power) and lipid peroxidation inhibition assays. The pro-oxidant property of compounds was measured as the rate of oxidation of Trolox. The Mg(II) and Mn(II)/Na(I) complexes with isoferulic acid showed higher antioxidant activity than ligand alone in DPPH (IFA, IC_50_ = 365.27 μM, Mg(II) IFA IC_50_ = 153.50 μM, Mn(II)/Na(I) IFA IC_50_ = 149.00 μM) and CUPRAC assays (IFA 40.92 μM of Trolox, Mg(II) IFA 87.93 μM and Mn(II)/Na(I) IFA 105.85 μM of Trolox; for compounds’ concentration 10 μM). Mg(II) IFA is a better scavenger of ^•^OH than IFA and Mn(II)/Na(I) IFA complex. There was no distinct difference in ABTS^•+^ and lipid peroxidation assays between isoferulic acid and its Mg(II) complex, while Mn(II)/Na(I) complex showed lower activity than these compounds. The tested complexes displayed only slight antiproliferative activity tested in HaCaT human immortalized keratinocyte cell line within the solubility range. The Mn(II)/Na(I) IFA (16 μM in medium) caused an 87% (±5%) decrease in cell viability, the Mg salt caused a comparable, i.e., 87% (±4%) viability decrease in a concentration of 45 μM, while IFA caused this level of cell activity attenuation (87% ± 5%) at the concentration of 1582 μM (significant at α = 0.05).

## 1. Introduction

**Figure 1:**
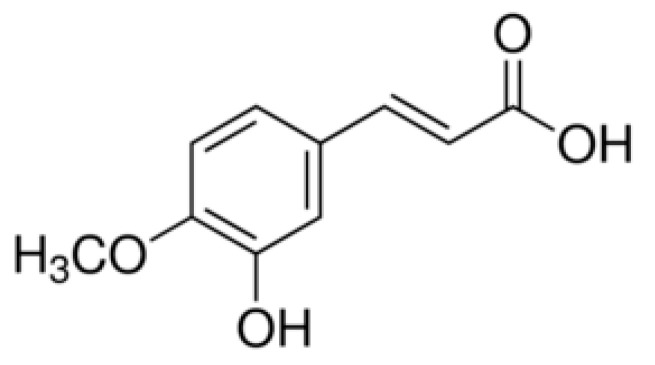
Chemical structure of isoferulic acid (IFA).

The main natural sources of antioxidants found in food are vegetables, fruits, legumes, oilseeds, grains, tea, herbs, and spices [1]. They contain mainly polyphenols (phenolic acids, flavonoids, anthocyanins), vitamin C, E, and carotenoids [2]. Due to the proven numerous beneficial effects of phenolic compounds over time, in recent years, there has been an increased interest in cinnamic acid derivatives as bioactive food ingredients [3]. Naturally occurring 3-hydroxy-4-methoxycinnamic acid (isoferulic acid, IFA) (Figure 1), the isomer of ferulic acid, belongs to the group of hydroxycinnamic acids and is known to possess different biological activities, including antioxidant, anticancer, antimicrobial, and antiviral (Table 1). The conducted studies reported the activity of isoferulic acid in combating diseases related to ROS (reactive oxygen species), such as diabetes (by lowering plasma glucose levels) [4,5], cardiovascular disease, coronary heart disease and heart attack [6]. Many of those mentioned diseases are associated with an irregularity in the reduction-oxidation (redox) balance in the body as a result of an increase in the concentration of free oxygen radicals such as superoxide anion radicals (O_2_^•−^) or hydroxyl radicals (^•^OH) [7]. Their excess in an organism may lead to damage of biologically relevant molecules such as proteins, lipids, polysaccharides, or nucleic acids and, as a consequence, cause homeostatic disruption and cell death [8]. The human body can counteract their excess by producing various endogenous antioxidant molecules or enzymes, such as superoxide dismutase, glutathione peroxidase, catalase, glutathione, ferritin, and uric acid [9]. However, the organism depends largely on the number of antioxidants supplied to the body through food and supplements (exogenous source) [10]. The numeric parameters describing the antioxidant, cytotoxic, antimicrobial, anti-diabetes, anti-inflammatory, antiviral, and other potential of isoferulic acid are shown in Table 1.

The ability to counteract oxidation reactions and neutralize free radicals by phenolic acids is directly related to the presence and location of hydroxyl groups linked to the benzene ring of the molecule. The antioxidant properties of phenolic compounds also expand with an increase in the number of –OH groups attached to the aromatic ring [28]. For example, *p*-coumaric acid, which has only one hydroxyl group in its structure, shows a lower ability to neutralize the DPPH^•^ radical than caffeic acid, which has two hydroxyl groups. It has been proven that hydroxycinnamic acids show higher antioxidant activity than hydroxybenzoic acids. This may be related to the presence of –CH = CH–COOH moiety, which provides a greater electron-donating ability than the -COOH group in hydroxybenzoic acids [29,30,31]. Increasing the stability of the phenoxyl radicals and antioxidant properties of the phenolic acids can be obtained by introducing a donor alkyl group electron or methoxy group (−OCH_3_) at the *ortho*-position [32]. Due to the placed methoxy group at the *ortho*- position to the hydroxyl group, ferulic acid is a more powerful antioxidant than *p*-coumaric acid [33]. The results obtained by Yi-Zhong Cai et al. [34] exhibited that substitution of the 3- or 4-hydroxyl group of caffeic acid by a –OCH_3_ group as in ferulic and isoferulic acids definitely improved the radical scavenging activity [34]. The presence of one or two methoxy groups in the ring of a phenolic compound containing only one hydroxyl group increases its antioxidant activity. The −OCH_3_ group, after substitution into the aromatic ring, shows properties that directly affect the charge distribution in the ring and, subsequently, the properties of the electron-donating OH groups [35]. However, the antioxidant activity of isoferulic acid has not yet been sufficiently measured, unlike other derivatives of cinnamic acids as, for example, ferulic, chlorogenic, or caffeic acid [18,36,37,38].

In recent years, the influence of metal ions on the antioxidant and antimicrobial properties of phenolic compounds (ligands) has become a topic of interest. Many papers report that flavonoids and phenolic acids in combination with metal ions may exhibit higher antioxidant activity than the ligands alone because of their ability to act as free radical acceptors [39,40,41]. For example, the complex of Zn(II) with curcumin protected the gastric mucosa against ethanol-induced injury more than curcumin alone [42]. In the work of Rubens and Wagner [43], complexation of rutin and galangin with Cu(II), Fe(II), Al(III), and Zn(II) ions increased their DPPH^•^ radical scavenging activity, i.e., the EC_50_ value for rutin was 18.23 μM, while for the complexes it was in the range of 5.02–3.76 μM [43]. Ca(II) complex of gentisic acid shows higher antioxidant activity in DPPH^•^ (2,2-diphenyl-1-picrylhydrazyl) radical, FRAP (ferric reducing antioxidant power), and CUPRAC (cupric reducing antioxidant capacity) assays than the ligand alone [44]. Moreover, it was proven that phenolic compound complexes with transition metals (such as Fe(II) or Mn(II)) often have higher bioavailability than pure ligands, e.g., quercetin shows increasing bioavailability in combination with Cu(II) ions [45]. Chelating of the ligand with metal ions may also affect the kinetics of the reaction and its acceleration [46]. There are many examples of Mg(II) and Mn(II) complexes with greater DPPH^•^ radical scavenging activity than the ligand alone, e.g., Mg(II) complexes with apigenin [47], luteolin [48], quercetin [49], Mn(II) complexes with luteolin [50], curcumin [51], and isatin hydrazine [52]. In addition, the higher antiradical properties of the metal complex of phenolic compounds compared with the free ligand can be explained by the acquisition of an additional radical scavenging metal center by the complex [43]. Investigation of the effect of complexation of phenolic compounds with metals can accelerate the development of new effective natural antioxidants that can be used in many areas, e.g., biotechnology, pharmacy, cosmetology, medicine, or the food industry.

In this study, magnesium and manganese were deliberately chosen as metallic centers in the synthesized complexes with isoferulic acid. Magnesium is the fourth most common cation found in the human body (after sodium, potassium, and calcium). It is required for numerous (over 300) enzymatic reactions in the body, such as protein synthesis, blood pressure regulation, blood sugar control, signal transduction, muscle, and nerve transmission [53,54,55,56]. Manganese is an essential microelement that is a part of several enzymes required for the proper synthesis of proteins, nucleic acids, and the cholesterol pathway [57,58]. Moreover, manganese plays a fundamental role in bone growth, regulation of blood sugar, blood coagulation, immune system and digestion, and defense against ROS [59]. Moreover, the extensive studies of the metal complexes with naturally occurring phenols with high antioxidant activity and well-described structure can be used in many areas, e.g., in the food, pharmaceutical, and cosmetics industries. High biological activity of several Mn and Mg complexes has been reported in the literature. For example, in the study of Vajragupta et al. [60], Mn(II)-curcumin complex exhibited a great capacity to protect brain lipids against peroxidation with IC_50_ of 6.3–26.3 μM. Manganese complexes with curcumin and its derivatives displayed high SOD-like catalytic activity [60]. Li et al. [61] described the medical potential of the Mg(II) complex of marbofloxacin (MB). In their research, MB-Mg complex showed twice as much antimicrobial activity than MB alone against *Staphylococcus aureus*, *Escherichia coli*, *Proteus species*, *Bacillus subtilis,* and *Micrococcaceae*. Moreover, the results of the acute toxicity test on mice showed that the complexation of MB with Mg(II) ions could probably decrease its acute toxicity [61]. Other studies found that magnesium chelation with isoorotic acid significantly increased its dissolution properties which may be useful in magnesium supplementation [62].

The studies devoted to structural properties and biological activity of isoferulic acid complexes are very limited. Therefore, in this work, the Mg(II) and Mn(II)/Na(I) complexes of isoferulic acid were synthesized and studied using FT-IR, FT-Raman, UV-VIS, and X-ray diffraction. These are the first crystal structures of the metal complexes of IFA reported in the literature. The anti-/pro-oxidant activity of the synthesized complexes was also studied and compared with the properties of ligand in the DPPH^•^, ABTS^•+^, CUPRAC, hydroxyl radical scavenging, lipid peroxidation, and Trolox oxidation assays. The cellular toxicity was tested against HaCaT human immortalized keratinocyte cell line.

## 2. Materials and Methods

### 2.1. Materials

All chemicals had an analytical purity and were used without further purification. Isoferulic acid, magnesium chloride (MgCl_2_), manganese(II) chloride (MnCl_2_), sodium hydroxide (NaOH), DPPH (2,2-diphenyl-1-picrylhydrazyl), ABTS (2,2-azino-bis(3-ethylbenzothiazoline-6-sulfonic acid), potassium persulfate (K_2_S_2_O_8_), copper(II) chloride (CuCl_2_), ammonium acetate (CH_3_COONH_4_), neocuproine (2,9-dimethyl-1,10-phenanthroline), iron(II) sulfate (FeSO_4_), salicylic acid (C_7_H_6_O_3_), trolox (6-hydroxy-2,5,7,8-tetramethylchroman-2-carboxylic acid), hydrogen peroxide (H_2_O_2_), phosphate buffer pH = 7, and horseradish peroxide (HRP) were purchased from Sigma-Aldrich Co. (St. Louis, MO, USA). Methanol was sourced from Merck (Darmstadt, Germany).

HaCaT human immortalized keratinocyte cell line (Thermo) has been chosen for the cellular toxicity test in mammalian cell culture [63]. Cells were cultured in DMEM medium supplemented with 4.5 g/L glucose, 2 mM L-glutamine, and 10% fetal bovine serum. For the cytotoxicity assessment of the formulations, an assay based on the neutral red (3-amino-7-dimethylamino-2-methylphenynosine hydrochloride) absorption capacity was selected [64]. Neutral red (N4638, Sigma Aldrich, Darmstadt, Germany) sterile solution in PBS (5 mg/mL) was added to the cell culture medium to a final concentration of 50 µg/mL and kept overnight at 37 °C before added to the cell culture. HaCaT cell line was obtained from CLS Cell Lines Service GmbH (Eppelheim, Germany). Fetal bovine serum was purchased from Gibco (Thermo Fisher Scientific, Inc., Waltham, MA, USA).

### 2.2. Synthesis

The Mg(II) complex of isoferulic acid was obtained as follows. First, 0.4 g of isoferulic acid was dissolved in 20.62 mL of the aqueous solutions of NaOH (0.1 M) in the stoichiometric molar ratio 1:1. Next, 2.06 mL of MgCl_2_ (0.5 M) was added to this solution in the stoichiometric molar ratio of 2:1 (ligand: Mg ion). The solution was mixed with the use of a mechanical shaker through 1 hour. After few days, the formed crystals were filtered and washed with the distilled water. The product reaction yield was 27%. The results of the elementary analysis for manganese(II) isoferulate gave the formula Mn_3_Na_2_(C_10_H_7_O_4_)_8_(H_2_O)_8_: %C = 50.51 (calc. %C = 50.56), %H = 4.60 (calc. %H = 4.66). The Mn(II)/Na(I) complex of isoferulic acid was obtained in a similar manner but with the use of MnCl_2_ (0.5 M) instead of MgCl_2_. For magnesium isoferulate, the formula was [Mg(H_2_O)_6_]⋅(C_10_H_9_O_4_)_2_⋅6H_2_O: %C = 38.27 (calc. %C = 38.32), %H = 6.64 (calc. %H = 6.75). The product reaction yield was 21%.

### 2.3. Single-Crystal X-ray Diffraction

The X-ray diffraction data for Mg(II) IFA and Mn(II)/Na(I) IFA were collected on an Oxford Diffraction Xcalibur CCD diffractometer (Abingdon, Oxfordshire, UK) using the graphite-monochromated MoKα radiation (λ= 0.7107 Å). The *CRYSALIS* [65] suite of programs was used for data collection, cell refinement and data reduction. A multi-scan absorption correction was applied. The structures were solved using direct methods implemented in *SHELXS-97* [66] and refined with the *SHELXL-97* program [66] (both operating under WinGX [67]). All non-H atoms were refined with the anisotropic displacement parameters. The hydrogen atoms in Mg(II) IFA were found in the difference Fourier maps and refined with the isotropic displacement parameters. In structure Mn(II)/Na(I) IFA, apart from the methoxy groups, the H-atoms attached to carbon were positioned geometrically and allowed to ride on the parent atoms with U_iso_(H) = 1.2 U_eq_(C). The remaining ones were located from the different Fourier maps and refined isotropically. The final data collection parameters and refinement statistics are summarized in Table 2. The molecular plots were drawn with Mercury [68].

The cif files for Mg(II) IFA and Mn(II)/Na(I) IFA were deposited at the Cambridge Crystallographic Data Centre as a Supplementary Material (CCDC 2079839-2079840). The copies of the data can be obtained free of charge on request: e-mail: deposit@ccdc.cam.ac.uk.

### 2.4. Spectroscopic Studies

The FT-IR spectra of the solid samples as KBr pellets were recorded on an Alfa Bruker spectrometer and analyzed in the range of 400–4000 cm^−1^. Raman spectra were recorded from 4000 to 400 cm^−1^ with a Multi-Raman (Bruker, Bremen, Germany) spectrophotometer. UV/VIS spectra of isoferulic acid and its complexes were recorded in the range of 200–400 nm using the UV/VIS/NIR Agilent Carry 5000 spectrophotometer (Santa Clara, CA, USA).

### 2.5. Anti-/Pro-Oxidant Study

The antiradical activity of the tested substances was determined against DPPH^•^, ABTS^•+^, ^•^OH, H_2_O_2_, and copper(II) reducing activity in CUPRAC assay. The assay with stable DPPH^•^ radical (2,2-diphenyl-1-picrylhydrazyl) was carried out according to the spectroscopic method described by Rice-Evans [69]. The samples reacted with the DPPH^•^ radical in a methanol solution for 1 h. After that time, the absorbance was measured by UV-VIS 5000 spectrophotometer ((Santa Clara, CA, USA) at 516 nm wavelength against methanol as blank. The percentage (%*I*) of DPPH^•^ radical scavenging activities of isoferulic acid and synthesized complexes were calculated using the following equation:

$$\%I=\frac{A_{control}^{516}-A_{sample}^{516}}{A_{control}^{516}}\times 100\%$$

where %*I* is the percent of inhibition of DPPH^•^ radical; $A_{control}^{516}$ is the absorbance of the control sample (only DPPH^•^ without tested substance); $A_{sample}^{516}$ is the absorbance of the tested sample. The concentration of the tested compounds was plotted against the percent of inhibition, and the IC_50_ values (the concentration of the antioxidant that is required to inhibit 50% of DPPH^•^ radical) were determined by linear regression analysis.

ABTS assay was performed according to Re et al. [70]. 2,2′-Azino-bis(3-ethylbenzothiazoline-6-sulfonic acid) diammonium salt (ABTS) and potassium persulfate (K_2_S_2_O_8_) were dissolved in distilled water to a final concentration of 7 mM and 2.45 mM, respectively. Then those two solutions were mixed in a volumetric ratio of 1:1 and left for 16 h at room temperature (23 °C) to produce ABTS cation radical (ABTS^•+^). After that, ABTS^•+^ solution was diluted with methanol in a volumetric ratio of 1:60 to obtain an absorbance of 0.700 at 734 nm. 1 mL of methanolic solution of the tested substance was added to 1 mL of diluted ABTS^•+^ solution, and the absorption reading was taken 7 min after mixing with the UV-VIS spectrophotometer. The absorbance was read at a 734 nm wavelength against methanol. The antiradical activity against ABTS^•+^ was presented as the percentage inhibition of ABTS^•+^ cation radicals (%I):

$$\%I=\frac{A_{control}^{734}-A_{sample}^{734}}{A_{control}^{734}}\times 100\%$$

where %*I* is the percent of inhibition of ABTS^•+^ radical; $A_{control}^{734}$ is the absorbance of the control sample; $A_{sample}^{734}$ is the absorbance of the tested sample.

Cupric reducing antioxidant activity (CUPRAC) assay was carried out according to [71] and is based on the reduction of Cu(II) ions to Cu(I) ions in the neocuproine complex by an antioxidant. Antioxidant properties were expressed as Trolox equivalents (μM) by using the calibration curve obtained for Trolox in the range of concentration 50–350 μM (y = 1.7302x + 0.0042; R^2^ = 0.9988).

Hydroxyl radical scavenging assay was determined according to [72]. The reaction mixture containing 1 mL of the tested compound (0.5 mM), 0.3 mL of FeSO_4_ solution (8 mM), 1 mL of salicylic acid ethanol solution (3 mM), and 0.25 mL of H_2_O_2_ (20 mM) was mixed and incubated at 37 °C. After 30 min of incubation, 0.5 mL of distilled water was added to the mixture, and the absorbance at 510 nm was read immediately. For the blank test, H_2_O was added instead of the tested sample, while H_2_O was used instead of H_2_O_2_ for the control sample. The ^•^OH radical scavenging activity was calculated as follows:

$$\%I=\left(1-\left(\frac{A_{control}^{510}-A_{sample}^{510}}{A_{blank}^{510}}\right)\right)\times 100\%$$

where $A_{sample}^{510}$ is the absorbance of the tested sample; $A_{control}^{510}$ is the absorbance of the control sample; $A_{blank}^{510}$ is the absorbance of the blank sample.

The linoleic acid peroxidation was carried out according to [73] with some modifications. Preparation of linoleic acid emulsion was made by mixing 1 mL of methanolic solution of the tested substance with 1 mL phosphate buffer pH 7.0 and 0.5 mL linoleic acid emulsion. The reaction mixture with methanol instead of the tested sample served as a control. All tested samples were incubated at 40 °C and measured every day for 5 days. Measurement of the peroxidation was performed by taking 0.1 mL of the incubated test sample solution and mixing it with 4.7 mL of 75% methanol and 0.05 mL 30% ammonium thiocyanate. After 3 min, 0.05 mL of 20 mM iron(II) chloride in 3.5% HCl was added. The absorbance was read immediately at a 500 nm wavelength against 75% methanol. Lipid peroxidation inhibition was calculated using the equation:

$$\%LPI=\frac{A_{control}^{500}-A_{sample}^{500}}{A_{control}^{500}}\times 100\%$$

where $A_{sample}^{500}$ is the absorbance in the presence of the tested sample, and $A_{control}^{500}$ is the absorbance of the control (without sample).

The pro-oxidant activity was measured as the rate of oxidation of Trolox according to the method described by Zeraik et al. [74]. In total, 0.5 mL of Trolox (C = 100 μM) was mixed with 0.5 mL of H_2_O_2_ (C = 50 μM), 0.5 mL of horseradish peroxide (C = 0.01 μM) in phosphate buffer (pH = 7), 0.05 mL of the tested substance (μM), and 0.495 mL of distilled water. The mixture was vortexed and incubated at room temperature. The control sample contained 0.05 mL of pure methanol instead of the tested substance. The absorbance measurements (at 272 nm) against the phosphate buffer were made every 10 min through 50 min.

All measurements were taken in 5 repetitions for 3 independent samples for each substance. The results were expressed as the mean of the values obtained for the replications. Average, standard deviation calculation, and graphs were performed with Microsoft Excel 2019.

### 2.6. Cell Viability Test

A day before the experiment, HaCaT cells were seeded in 96-well plates at a density of 10,000 cells per well in 100 μL of DMEM medium. Isoferulic acid (IFA) and its complexes were dissolved in DMSO to the highest concentrations (6.2 mg IFA ad 200 µL, 6 mg Mn(II)/Na(I) IFA ad 2000 µL, and 5.7 mg Mg(II) IFA ad 2000 µL). Next, the obtained DMSO solutions were mixed with cell culture medium in the proportion of 1:100 to obtain the highest working drug solutions. The DMSO content in the cell culture medium did not exceed 1%. These solutions were further diluted by factor 2 to obtain a decreasing concentration of the compounds. Next, the cell medium from the 96-well plate cultures was replaced with the working solutions of the IFA or its complexes and cells were incubated for 24 h. After that time, the medium was removed, and cells were incubated for 4 h in a medium containing 50 µg/mL of neutral red (NR). The NR-containing medium was removed, and the cultures were rinsed twice with phosphate-buffered saline (PBS). Cells were treated with a solution of 50% alcohol and 1% acetic acid in water to lyse the cells and dissolve the absorbed dye. The preparations were mixed on a shaker for 10 min, and the fluorescence was measured at excitation and emission wavelengths of 530 nm and 645 nm, respectively. Experiments were performed in 4–6 repetitions. As a negative control (background), cell culture-free wells were measured. As a positive control, wells containing untreated cell cultures were measured.

## 3. Results and Discussion

### 3.1. Molecular and Crystal Structure

**Figure 2:**
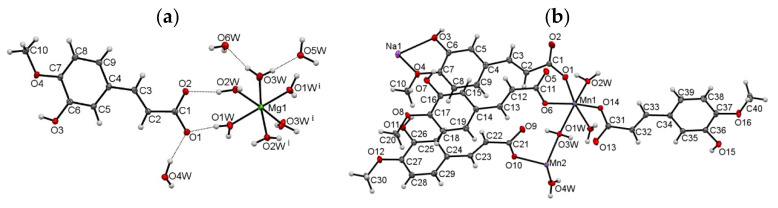
The asymmetric unit of: (**a**) [Mg(H_2_O)_6_]⋅(C_10_H_9_O_4_)_2_⋅6H_2_O) and (**b**) [Mn_3_Na_2_(C_10_H_7_O_4_)_8_(H_2_O)_8_]*_n_* with the atom-labeling scheme. Displacement ellipsoids are drawn at the 50% probability level. Dashed lines indicate hydrogen bonds. Symmetry code: (i) -x, y, -z +1/2.

Single-crystal X-ray diffraction studies on the Mg(II) and Mn(II)/Na(I) complexes of isoferulic acid confirmed their chemical composition, as determined from the elemental analysis and spectroscopy studies. The relevant geometric parameters for the structures are given in Table S1 (Supplementary Information). The molecular plots with the atom numbering schemes are presented in Figure 2.

The data revealed that compound Mg(II) IFA crystallizes in the monoclinic space group *C*2/*c*, with half of Mg(II) cation, one isoferulic acid anion, three coordinated and three lattice water molecules in the asymmetric part of the unit cell. The metal ion occupies a special position on the twofold axis. The studied compound is an ion-pair metal complex. The cationic part consists of one Mg(II) ion and six water molecules which fill the first coordination sphere of the metal center, giving the [Mg(H_2_O)_6_]^2+^ complex with the slightly distorted octahedral geometry (Figure 2b). The Mg–O_w_ distances are in the range of 2.042(1)–2.098(1) Å, typical of an Mg(II) center bound to oxygen donor atoms (CSD, Version 5.42) [75]. The distortion from an ideal octahedron manifests itself through the nonlinear O1w–Mg1–O1w^(i)^ and O2w–Mg1–O3w^(i)^/O2w^(i)^ –Mg1–O3w axes of 171.81(6)° and 172.66(4)°, respectively (Table S1, Figure 2a). The remaining O–Mg1–O angles vary in the range 85.07(4)–97.23(4)°. All coordinated water molecules are terminal ligands. The isoferulate ion adopts the expected *trans* configuration around the C2 = C3 double bond. The aliphatic and aromatic moieties are almost co-planar, as shown by the dihedral angle between the best planes of the phenyl ring and carboxylate group, being 9.6°. A small distortion is mostly due to a slight rotation around C3–C4 and C1–C2 bonds (Table S1). The hydroxy and methoxy substituents stay in the plane of the phenyl ring; the planarity of that system is additionally stabilized by the intramolecular O3–H3⋅⋅⋅O4 hydrogen bond. The C2 atom is *syn*-oriented with respect to the aromatic C5 atom (Figure 2a). The overall conformation of the ligand is similar to that of ferulic acid [76] and ferulate ion in ammonium ferulate monohydrate [77]. However, it is in contrast with that observed in sodium salt of ferulic acid [78], where the C2 and C5 atoms are *anti* to each other. The C–O distances in -COO^−^ are almost the same (1.271(1), 1.277(1) Å), which confirms complete deprotonation of the carboxylic group.

**Figure 3:**
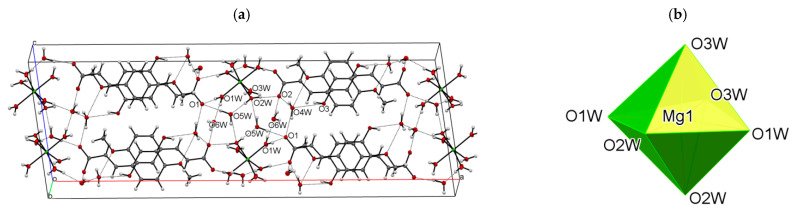
(**a**) The alternating cationic and anionic (100) layers in Mg(II) IFA, viewed along the *b* axis. Dashed lines indicate hydrogen-bonding interactions. (**b**) The polyhedral representation of [Mg(H_2_O)_6_]^2+^ cation.

In the crystal lattice, the ligand molecules are organized into polar monolayers parallel to the (100) crystallographic plane. The two-dimensional (2D) architecture is stabilized by chelating C8–H8⋅⋅⋅O3, C10–H10c⋅⋅⋅O3 (Table S2) hydrogen bonds between the adjacent *c*-glide plane related molecules. The additional stabilization is provided by the π-stacking contacts (*d*_(C4__⋅⋅⋅C6)_ = 3.346(2) Å) between the overlapping aromatic rings. The [Mg(H_2_O)_6_]^2+^ cations and lattice water molecules fill in the space between the anion layers. The carboxylate groups, as well as the hydroxy and methoxy substituents, oriented outside these layers, participate in the formation of numerous strong hydrogen bonds with the coordinated and lattice water molecules (Table S2, Figure 3a).

**Figure 4:**
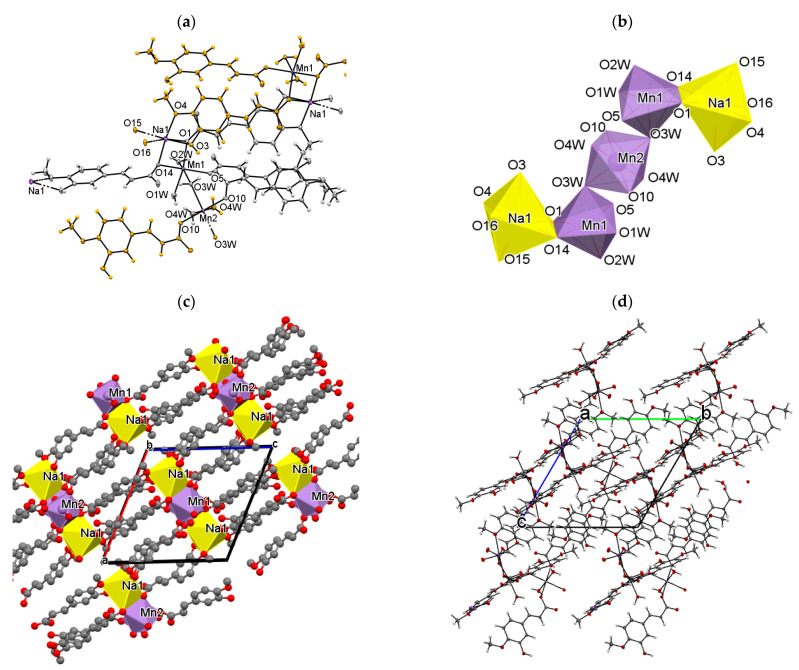
The part of the crystal structure of Mn(II)/Na(I) IFA presenting: (**a**) the coordination environment of Mn and Na cations in the crystal [Mn_3_Na_2_(C_10_H_7_O_4_)_8_(H_2_O)_8_]*_n_* (the asymmetric unit is marked grey); (**b**) coordination polyhedra of Mn1, Mn2, and Na1 ions; (**c**) crystal packing view down the *b* axis with polyhedral representation of the metal centers; (**d**) the 3D framework in view along the *a* axis.

The chemical formula of Mn(II)/Na(I) complex of isoferulic acid, as obtained from the single-crystal X-ray data, is [Mn_3_Na_2_(C_10_H_7_O_4_)_8_(H_2_O)_8_]*_n_*. The compound crystallizes in the triclinic space group *P*-1. The asymmetric unit of the crystal (Figure 2b) comprises one and a half Mn(II) cations, one Na(I) ion, four IFA anions, and four coordinated water molecules. One of the Mn(II) ions is located on the inversion center. The diffraction studies revealed that the compound is a 3D coordination polymer. The centrosymmetric pentanuclear subunits can be distinguished in the resulting covalent framework. As shown in Figure 4a,b, each metal center is six-coordinated. The coordinating oxygen atoms are derived from both organic and water molecules. The Mn1 cation is ligated by three carboxylate oxygen atoms (O1, O5, O14) and three water molecules, all of them coming from different (symmetrically independent) ligand moieties. The Mn2 ion, which occupies the special position, is coordinated by four water molecules (O3w, O4w) and two symmetry equivalent, monodentate carboxylate oxygen atoms (O10). In turn, the coordination environment of Na1 cation is formed by two carboxylate oxygen atoms (O1, O14), two hydroxy (O3, O15), and two methoxy (O4, O16) oxygen atoms from four isoferulate ions. Two ions coordinate the Na1 atom in a monodentate way (through carboxylate oxygen atoms), two others chelate the metal center via the hydroxy and methoxy substituents (Figure 4a). It is worth noting, similar chelating behavior of the ligand was observed in the crystal of sodium ferulate [78]. The Mn−O_carb_ and Mn−O_w_ bond lengths, being in the range of 2.141(1)–2.172(2) Å and 2.140(2)–2.288(2) Å (Table S1), respectively, are in good agreement with those observed for the other six-coordinated Mn(II) complexes with the O-donor atoms [79]. The Na−O distances vary from 2.374(2) to 2.514(2) Å and are well within the expected values for Na−O*_carb_* and Na−O_w_ bond distances [75]. The differences in the bond lengths cause significant distortion of the coordination polyhedra of Mn1 and Na1. The angles, which are 90° in the regular octahedron, in this case, range from 81.62(6)° to 102.69(6)° (Mn1) and from 64.82(5)° to 121.07(6)° (Na1). The remaining ones, which should be equal to 180°, are 163.32(6)–169.23(6)° and 153.36(6)–174.40(6)°, respectively.

The organic molecules exhibit two types of coordination modes in the studied structure. Two out of four symmetry independent moieties play the role of monodentate ligands, the remaining ones (C1 >> C9 and C31 >> C39) are of tetradentate character. In the latter, the carboxylate groups coordinate in a bidentate fashion, bridging two adjacent Na1 and Mn1 metal centers in the μ^2^ −η^2^:η^0^ mode (Figure 4a). Among the water molecules, three are monodentate terminal ligands, whereas the fourth one (O3w) coordinates in a bidentate fashion and serves as a bridge between two symmetrically independent Mn(II) ions (Figure 4a).

In spite of the different chemical environments, the bond lengths, angles, and torsion angles in the symmetry-independent ligands are quite similar, and they do not deviate significantly from those observed in complex Mg(II) IFA. The only exception is the molecule C1 >> C10, which exists as an *anti*-conformer, regarding the relative orientation of aliphatic C2 and aromatic C5 atoms (Figure 2b). Interestingly, there is also a visible difference in the C−O bond lengths (1.229(3) and 1.290(3) Å) within the carboxylate group in this ligand, while in the remaining ones, the C−O_free_ and C−O_coord_ distances are comparable (Table S1).

As shown in Figure 4b, the coordination polyhedra of Mn1 and Na1 ions, which share the edges, are connected further with the Mn2 polyhedron via O3w corners, giving centrosymmetric pentanuclear subunits (Figure 4c). The metal-to-metal distances within the metallic five-membered Na1−Mn1−Mn2−Mn1−Na1 chain, running almost parallel to the (−101) crystallographic plane, are Mn1⋅⋅⋅Mn2: 4.073(2) Å and Na1⋅⋅⋅Mn1: 3.489(2) Å. The adjacent metallic chains are linked via the organic ligands giving the complex 3D covalent framework (Figure 4d). Additional stabilization of the structure is provided by the extensive net of relatively short, directional O−H⋅⋅⋅O hydrogen bonds (Table S2, SM). Each water molecule plays the role of a double proton donor, and each hydoxyl group is a single donor and a single acceptor in those interactions. It is worth noting that all carboxylate oxygen atoms, also those involved in coordination to the metal centers, serve as proton acceptors, whereas all methoxy O-atoms are excluded from the H-bonding interactions.

### 3.2. Spectroscopic Studies

The obtained FT-IR and FT-Raman spectra of isoferulic acid and its complexes with Mg(II) and Mn(II)/Na(I) ions are displayed in Figures S1 and S2. The wavenumbers, intensity, and assignment of selected bands from the spectra are gathered in Table 3. In the FT-IR spectrum of isoferulic acid, characteristic bands assigned to the stretching vibrations of the carbonyl group ν(C=O) were found at the wavenumbers: 1694 and 1671 cm^−1^. Moreover, the bands derived from the out-of-plane vibrations of the carboxylic group: γ(OH)_COOH_ at 949 cm^−1^ and γ(C=O) at 571 cm^−1^ occurred. The spectra of the synthesized complexes show characteristic bands resulting from the vibrations of the carboxylate anion, i.e., asymmetric ν_as_(COO^−^) and symmetric ν_s_(COO^−^) stretches, located, respectively, in the region of 1556–1552 cm^−1^ and 1413–1402 cm^−1^ (IR) (Raman: 1413–1410 cm^−1^). In the infrared spectra of the complexes, there is also a band corresponding to the symmetric vibrations in-the-plane β_s_(COO^−^) in the range 862–860 cm^−1^ (Raman: 880-874 cm^−1^). Different locations of bands assigned to the vibrations of the ring −OH group can be noticed in the spectra of studied compounds. In the FT-IR spectrum of isoferulic acid and Mn(II)/Na(I) IFA, the stretching vibrations of the −OH substituent is at 3406 and 3412–3171 cm^−1^, respectively. Conversely, in the spectra of Mg(II) IFA, the bands derived from the stretches of -OH are located in the range 3549–3415 cm^−1^. The band assigned to ν(O-CH_3_) stretches is located at 1024 and 1025 cm^−1^, respectively in the FT-IR spectra of acid and Mn(II)/Na(I) IFA, whereas, in the spectra of Mg(II) IFA, this band is shifted to higher wavenumber: 1031 cm^−1^ (FT-IR) and 1038 cm^−1^ (FT-Raman). It suggests that in the case of isoferulic acid and Mn(II)/Na(I) IFA, the −OH and −OCH_3_ substituents in the ring take part in the intermolecular hydrogen bonding (in the case of isoferulic acid) or in sodium-ion bonding (in Mn(II)/Na(I) IFA molecule). Moreover, in the spectra of studied compounds, there are bands resulting from the vibrations of the aromatic ring. Most of the bands in the spectra of the metal complexes derived from the aromatic ring shift towards higher wavenumbers in comparison with the spectrum of ligands. This indicates a stabilization of the aromatic system as a result of bond length equalization and the increase in the force constants of the bonds.

The UV/VIS absorption spectra of the studied compounds were recorded in methanol and demonstrated in Figure S3. In the UV/VIS spectrum of isoferulic acid (0.01 mM), three maxima of absorption occurred at 322, 294, and 243 nm (Table 4). These bands are related to the π → π* electronic transitions in the aromatic ring. The maxima of absorbance in the spectra of the complexes were slightly shifted towards lower wavelengths (hipsochromic shift). In the UV spectrum of Mg(II) IFA (0.01 mM), these maxima occurred at 316, 239, and 291 nm and in the case of Mn(II)/Na(I) IFA (0.01 mM) at 315, 290, and 235 nm.

### 3.3. Anti-/Pro-Oxidant Study

**Figure 5:**
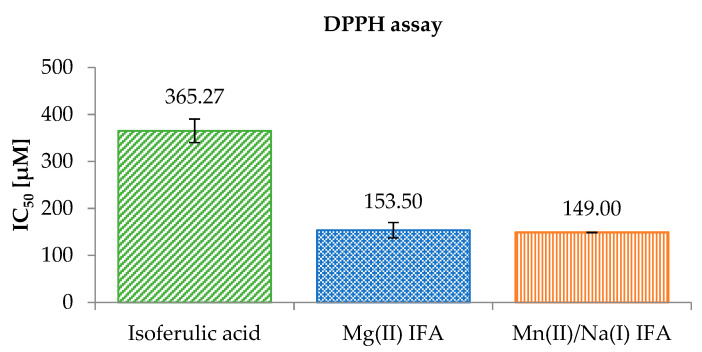
A comparison of the antioxidant activities of the tested compounds (methanolic solutions) measured by DPPH^•^ assay.

IC_50_ parameter is the concentration of antioxidant that is required to inhibit 50% of the DPPH^•^ free radicals. The lower value of IC_50_ means that the substance had a higher antioxidant activity. The results of reactions of studied phenolic compounds with DPPH^•^ radicals (Figure 5) showed that Mg(II) IFA (IC_50_ = 153.50 ± 16.26 μM) and Mn(II)/Na(I) IFA (IC_50_ = 149.00 ± 0.57 μM) had much higher antiradical activity than the ligand alone (IC_50_ = 365.27 ± 24.96 μM). The antiradical properties of tested phenolic compounds increased in the following order: IFA < Mn(II)/Na(I) IFA < Mg(II) IFA. This suggests that the used metal ions could significantly change the biochemical properties of the isoferulic acid.

In our study, the IC_50_ for isoferulic acid was 365.27 μM, but in the literature different values of the IC_50_ for IFA were reported: 40.20 mM [14], %I ≈ 10% (at the concentration of 25 μM) [80], and %I = 2.9, 13.2, 25.8, 95.3, and 95.4 (for IFA concentrations of 10, 50, 100, 500, and 1000 μg/mL, respectively) [81]. Various experimental conditions, such as reaction period, the type of the solvent, and the pH of the reaction mixture have an influence on the ability of antioxidants to scavenge the DPPH^•^ radical [82,83,84]. Nenadis and Tsimidou [85] reported that in the DPPH^•^ test performed, the %RSA (level of the relative activity) for isoferulic acid in ethanol as a solvent was 3.5%, while in acetonitrile and tert-butylalcohol were 1.8 and 1.2%, respectively [85].

The ABTS and CUPRAC assays were carried out for two concentrations of studied compounds (10 and 20 μM). The results showed that IFA and Mg(II) IFA(at both concentrations) had similar results of the ABTS^•+^ radical inhibition (Table 5). Mn(II)/Na(I) IFA was the weakest scavenger of ABTS^•+^ among the tested compounds (%I= 51.95% and 58.91%). Cupric reducing antioxidant activity (CUPRAC) assay showed the same relationship as the DPPH^•^ radical test. Isoferulic acid had the weakest ability to reduce copper(II) ions comparing with the synthesized complexes (in two different concentrations 10 and 20 μM, the CUPRAC values for IFA: 40.92 ± 3.79 and 46.31 ± 5.90 μM of Trolox). Moreover, we observed that in the CUPRAC assay, the antioxidant activity of the synthesized complexes increased proportionally with the increase in their concentration. In the case of isoferulic acid, such a relationship was not noticed.

Depending on the type of reaction mechanism, the above tests can be divided into two types. The CUPRAC assay is based on the reaction of a single electron transfer (SET) from an antioxidant to a radical. While the DPPH^•^ and ABTS^•+^ radical assays are based on a mechanism involving two types of reaction simultaneously—HAT (Hydrogen Atom Transfer) and SET. The studies of [30,31,32] revealed that tests based on the HAT-type reaction mechanism are dependent on the ionization potential (IP) of studied compounds, i.e., the minimum amount of energy needed to detach an electron from a molecule or atom and from the bond dissociation energy of the group that is the donor of the hydrogen atom. In addition, the type of solvent and the pH of the environment do not affect HAT-type reactions, unlike the SET reaction, which is dependent on the acidity of the environment [86,87,88]. Despite the fact that the DPPH^•^ and ABTS^•+^ radicals assays are based on the same type of reaction mechanism (HAT and SET), different results were obtained in these experiments. Different kinetics between tested substances and these radicals probably affect the course of these reactions. Moreover, the lower solubility of tested compounds in water solution in the case of ABTS^•+^ assay may influence the final results (in the experiment with DPPH^•^ was carried out in methanol). The results obtained in the DPPH^•^ and CUPRAC assays for the tested compounds showed higher antioxidant activity of the isoferulic complexes than ligand alone. In the ABTS^•+^ cation radical assay, the inverse relationship was obtained.

**Figure 6:**
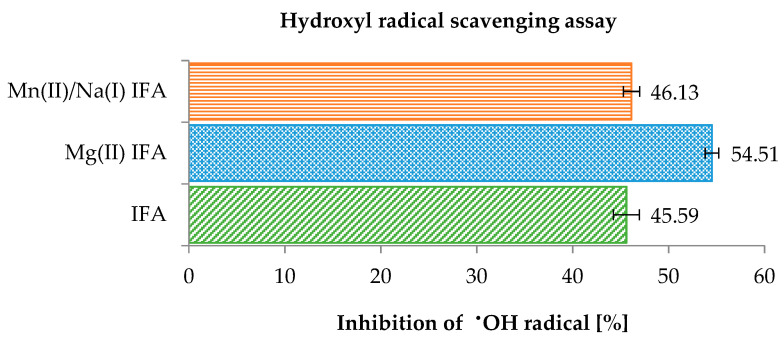
A comparison of the antioxidant activities of tested compounds (0.5 mM) measured by hydroxyl radical scavenging assay.

The hydroxyl radical is considered to be one of the most reactive species of oxygen (ROS) produced in chemical and biological systems. It can be generated by Fenton reaction (Fe(II) + H_2_O_2_ → Fe(III) + OH^−^ + ^•^OH) [89]. In this study, hydroxyl radical scavenging activity was measured as the ability of the studied compounds at a concentration of 0.5 mM to scavenge the ^•^OH radicals. The results of the analysis are presented in Figure 6. In the hydroxyl radical scavenging assay, the percentage of ^•^OH radical inhibition was equal to 45.59% for isoferulic acid and was in the range of 46.13% to 54.51% for synthesized complexes. The hydroxyl radical scavenging ability of tested compounds increased as follows: IFA ≤ Mn(II)/Na(I) IFA < Mg(II) IFA.

**Figure 7:**
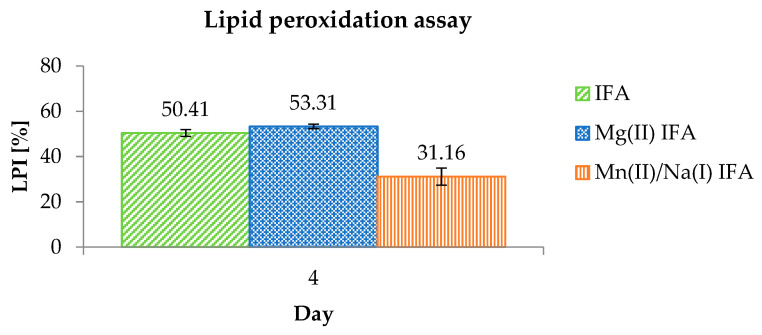
The antioxidant activity of the tested compounds (0.5 mM) measured in the lipid peroxidation assay on the fourth day of measurement.

The antioxidant properties of the studied compounds were also determined by measuring the lipid peroxidation inhibitory capacity. This method is based on the reaction of iron(II) cations with the products of linoleic acid peroxidation. As a result, iron(III) ions are formed, which, together with thiocyanate ions, give a red complex color observed at λ = 500 nm wavelength [73]. Among the tested compounds, Mg(II) IFA showed the highest inhibition value of lipid peroxidation (53.31 ± 0.96%) on the fourth day of measurement (Figure 7). A similar result was obtained for the isoferulic acid (50.41 ± 1.49%). Mn(II)/Na(I) IFA inhibited the lipid peroxidation in 31.16 ± 3.79%.

**Figure 8:**
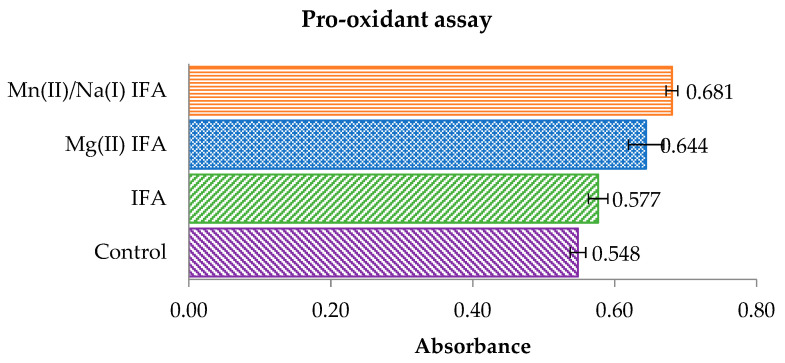
A comparison of the absorbance (at 272 nm) of Trolox quinone (concentration of tested compounds 0.5 mM) at time 0 in the pro-oxidant assay.

**Figure 9:**
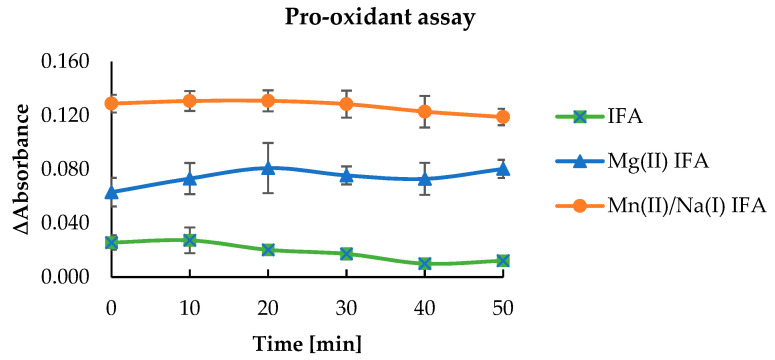
Comparison of the difference in absorbance of tested compounds (0.5 mM) on the rate of the Trolox oxidation reaction measured every 10 min for 50 min.

The pro-oxidant properties of isoferulic acid and tested complexes were defined as a degree of Trolox oxidation. The radicals of IFA and the metal complexes are produced in their reaction with H_2_O_2_ catalyzed by horseradish peroxide. Then, the phenoxyl radicals react with Trolox, which undergoes oxidation to Trolox radicals and then Trolox quinones. The phenoxyl radical is transformed into phenolic compounds. The maximum absorption for Trolox quinone is 272 nm [74]. Figure 8 shows the results of absorbance measurement for the tested samples and control in time 0 (at the beginning of the experiment). After each measurement, the difference in absorbance between the tested samples and the control was counted. The absorbance difference results obtained after 50 min are shown in Figure 9. The Mg(II) and Mn(II)/Na(I) isoferulic acid complexes increased the rate of Trolox oxidation compared with the free ligand. After 50 min, the difference in absorbance of the tested complexes was 0.119 ± 0.006 (Mn(II)/Na(I) IFA), 0.080 ± 0.007 (Mg(II) IFA), and for isoferulic acid, the ΔAbs. was 0.012 ± 0.003.

In our study, the complex of isoferulic acid with metal ions had significantly higher antioxidant activity compared to alone IFA in DPPH^•^ and CUPRAC assays. Multiple reports in the literature have shown that the complexation of phenolic compounds with metal ions may increase biological activity and bioavailability of the parent ligand [42,43,44,45], but there are also many studies describing a decrease in ligand activity due to the complexation with metal ions [90]. The higher biological activity of metal complexes compared with ligand was reported by other authors as well. For example, Co(II), Cd(II), and Cu(II) complexes of quercetin showed a greater ability to inhibit DPPH^•^ radical than the pure ligand [91,92]. Complexation with Mg(II) ions increased the hypoglycemic and antibacterial activity compared to luteolin alone, especially against *E. coli* [50]. Kostyuk et al. [93] reported that chelation of rutin, epicatechin, and dihydroquercetin with Fe(II), Fe(III), Cu(II), or Zn(II) cations increased their effectiveness of radical scavenging, improved their protective activity against cell damage caused by asbestos compared with uncomplexed flavonoids [93]. It has been determined that the coordination of metal cations in the compound significantly changes the electronic charge distribution in the cation-binding sites. Moreover, it can be assumed that the antioxidant activity of the ligand depends on the type of metal cation in the complex [94]. Metal ions Mg(II) and Mn(II) studied in the above work are characterized by a high ionic potential, thanks to which they can stabilize the electron density distribution of a ligand and thus increase its antioxidant properties. Depending on the concentration and the ratio of the amount of phenolic compound to the metal ion, complexes can possess anti- or pro-oxidant properties [95]. The mechanism of pro-oxidative action based on the presence of metal cations consists in the generation of free phenoxyl radicals, which, e.g., may lead to damage in the structure of DNA or acceleration of lipid peroxidation [96].

### 3.4. Cell Viability

**Figure 10:**
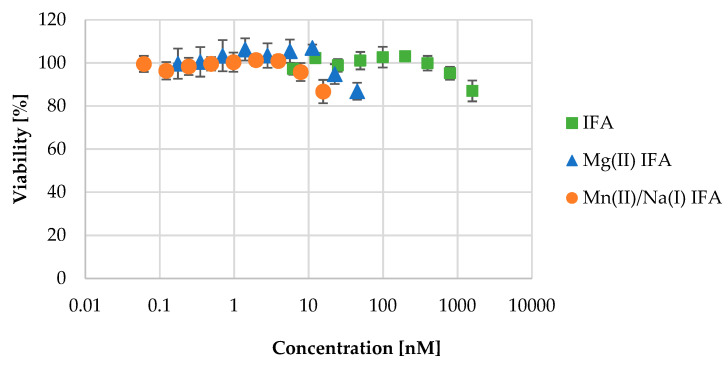
Cell viability assay test results for IFA (0–1.582 μM), Mg(II) IFA (0–45 μM), and Mn(II)/Na(I) IFA (0-16 μM). The error bars represent standard deviation values.

The results of the influence of the tested compounds on cell viability were estimated by the neutral red uptake test, a popular cytotoxicity test with many biomedical and environmental applications [64]. It is based on the ability of viable cells to incorporate and bind the supravital dye neutral red in the lysosomes. According to this assay, the tested complexes displayed slight antiproliferative activity within the solubility range (Table 6). As seen in Figure 10, all compounds caused a decrease in HaCaT cell viability, however at different concentrations, e.g., 16 μM for Mn(II)/Na(I) IFA and 45 μM for Mg(II) IFA, while IFA caused this level of cell activity attenuation at the concentration of 1 582 μM. It can be concluded that complexing IFA to metals strongly increases the toxicity of the obtained compound, but at the same time, it strongly decreases its availability in the water-based medium.

## 4. Conclusions

The Mg(II) and Mn(II)/Na(I) complexes of isoferulic acid were synthesized and studied by FT-IR, FT-Raman, UV/VIS, and single-crystal X-ray diffraction.

The structural studies revealed that the isoferulic acid is a versatile ligand with diverse coordination modes. The crystallographic data and vibrational spectra showed that in the case of the Mn(II)/Na(I) complex, metal ions were coordinated by carboxylate O-atoms and the additional coordination by −OCH_3_ and −OH substituents occurred. The changes in the location of bands assigned to the aromatic system and vinyl group -C=C- in the spectra of ligand and complexes were caused by the effect of metal ions on the electronic charge distribution of molecule, which consequently can lead to an increase in the electron or hydrogen atom donor properties and stability of phenoxyl radicals. The anti-/pro-oxidant activity of the studied compounds was measured by the use of DPPH^•^, ABTS^•+^, CUPRAC, hydroxyl radicals scavenging, pro-oxidation, and lipid peroxidation assays. The results of this study indicate that the complexes of isoferulic acid have higher antioxidant properties than ligand alone. The Mg(II) and Mn(II)/Na(I) complexes with isoferulic acid are better DPPH^•^ and ^•^OH radical scavengers and reveal higher cupric reducing antioxidant activity than the ligand alone. In ABTS^•+^ and lipid peroxidation assays, isoferulic acid and its Mg(II) complex revealed similar antioxidant properties, whereas Mn(II)/Na(I) complex showed the lowest ABTS^•+^-scavenging activity. Additionally, the complexation of isoferulic acid with Mg(II) and Mn(II)/Na(I) metal ions increased the pro-oxidant activity of the molecules compared to the ligand alone. Metal complexation caused a pronounced increase in the toxicity of the compounds, and the Mn(II)/Na(I) complex showed the highest toxicity in the keratinocyte HaCaT cell line.

## Figures and Tables

**Table 1:** Biological activity of isoferulic acid.

Antioxidant/Method; Parameter	Ref.
DPPH^•^; IC_50_ = 4.58 μg/mL; EC_50_ = 5.65 μM; EC_50_ = 0.75 μM; IC_50_ = 40.20 mM	[11,12,13,14]
ABTS^•+^; IC_50_ = 1.08 μg/mL	[11]
FRAP; IC_50_ = 8.84 μg/mL; FRAP value = 53.83 mM Fe^2+^; FRAP value = 4.38 mM Fe^2+^	[11,13,15]
CUPRAC; IC_50_ = 7.69 μg/mL	[11]
^•^OH; IC_50_ = 1.57 μg/mL	[11]
O_2_^•−^; IC_50_ = 13.33 μg/mL	[11]
Anti-lipid peroxidation; IC_50_ = 7.30 μg/mL	[11]
Cytotoxic	
the inhibition of cell viability, inducing cell apoptosis and triggered cell cycle arrest in G2/M phase in hematologic cancer cell lines (Raji, K562 and Jurkat)	[16,17,18]
Antimicrobial	
antibacterial activity against *Staphylococcus aureus*, *Bacillus subtilis*	[19]
antifungal activity against *Candida albicans*, *Aspergillus fumigatus*, *Aspergillus flavus,* and *Aspergillus niger*	[19,20]
Anti-diabetes	
lowering plasma glucose levels	[4,5]
antiglycation action against aldehyde-associated glycation in high-density lipoprotein (HDL)	[21]
protection against human serum albumin (HSA) structural changes induced by ROS and glycation	[13]
activation of α_1A_-AR adrenoceptor by IFA increasing the glucose uptake via PLC–PKC pathway in cultured myoblast C_2_C_12_ cell of mice	[22]
protection against fructose- and glucose-mediated glycation in vitro	[23]
Anti-inflammatory	
reducing joint pain, swelling, and inflammation	[24]
inhibition of the production of pro-inflammatory cytokines IL-6, TNF-α, and IFN-γ in lipopolysaccharide (LPS)-stimulated human whole blood	[25]
Antiviral	
elimination of viral infections, e.g., the production of macrophage inflammatory protein-2 (MIR-2) in a murine macrophage cell line, in response to respiratory syncytial virus (RSV) infection	[26]
Other properties	
repellent activity against the stored grain pest *Tribolium castaneum*	[27]

**Table 2:** Crystallographic data and final structure refinement details for the studied complexes.

	Mg(II) IFA	Mn(II)/Na(I) IFA
Formula	MgC_20_H_42_O_20_	Mn_3_Na_2_C_80_H_88_O_40_
Formula weight	626.85	1900.30
T (K)	100(2)	100(2)
Crystal system	Monoclinic	Triclinic
Space group	*C*2/*c*	*P*-1
*a* (Å)	35.329(3)	12.827(1)
*b* (Å)	6.9547(4)	13.425(1)
*c* (Å)	12.162(2)	14.566(1)
*α* (°)	90	116.37(1)
*β* (°)	97.21(1)	108.22(1)
*γ* (°)	90	95.81(1)
*V* (Å^3^)	2964.6(6)	2047.8(3)
*Z*	4	1
*d*_calc_ (g cm^−3^)	1.404	1.541
*θ* range (°)	2.99–27.48	2.81–27.48
*µ* (mm^−1^)	0.145	0.565
*R*_int_	0.032	0.028
Refl. collected/unique/obs. [*I*>2*σ*(*I*)]	11134/3389/2059	16224/9369/7824
Parameters/restrains	270/0	661/0
Completeness to θ_max_	0.999	0.998
*R*_1_, *wR*_2_ [*I*>2*σ*(*I*)]	0.0328; 0.0861	0.0404; 0.0944
*R*_1_, *wR*_2_ [all data]	0.0411; 0.0918	0.0518; 0.1020
GOF on F^2^	1.056	1.092
Max. and min. residual density (e Å^−3^)	0.31; −0.22	0.45; −0.31

**Table 3:** Wavenumbers (cm^−1^), intensities, and assignments of the selected bands occurring in the IR and Raman spectra of isoferulic acid, Mg(II) IFA, and Mn(II)/Na(I) IFA.

Isoferulic Acid		Mg(II) IFA		Mn(II)/Na(I) IFA		Assignments
IR	Raman	IR	Raman	IR	Raman	
3406s *		3474vs 3463vs 3415vs 3238s		3412s 3171s		ν(OH)_ar_
3078vw						ν(OH)_COOH_
2943w	2945vw	2934m	2935vw	2944s	2944vw	ν(CH)
2848m	2848vw	2841m	2844vw	2838s	2847vw	ν(CH)
1694s 1671s						ν(C=O)
1629s	1635vs	1639s	1632m	1636vs	1634vs	ν(CC)_-C=C-_
1613s	1615s	1617s 1595m	1615vs	1593s	1608m	ν(CC)_ar_
		1556s		1522vs		ν_as_(COO^−^)
1538m 1513vs		1532s				ν(CC)_ar_
1454m	1468vw	1466w	1462vw			δ_as_(CH_3_)
1443m		1432m	1440vw	1440vs	1438vw	ν(CC)_ar_
		1413s	1413w	1402vs	1410w	ν_as_(COO^−^)
1323s		1300m	1312vw		1311vw	ν(C-O)
1265vs	1279m	1253m	1275s	1253vs	1277m	β(CH) + ν(C-O)
1136s	1137w	1126s	1138vw	1136vs	1138vw	β(CH)
1024m		1031m	1037vw	1025s		ν(O-CH_3_)
977m	977vw	974m	980vw	981s	985vw	γ(CH)_-C=C-_
949m						γ(OH)_COOH_
		860w	880vw	862s	874vw	β_s_(COO^−^)
817m	818vw	816m		810s		γ(CH)_ar_
		599m		602m		γ_s_(COO^−^)
571w						γ(C=O)
		575m	566vw	565s		β_as_(COO^−^)
521m		535m	512vw	513m		βO-(CH_3_)
448vw		477m		450w		φ(CC)

* ν—stretching vibrations, δ—deforming in plane and out-of-plane bending vibrations, β—in-plane bending modes, γ—out-of-plane, φ—the aromatic ring out-of-plane bending modes, α—the aromatic ring in-plane bending modes.

**Table 4:** The wavelengths of maximum absorbance from the UV/VIS spectra of isoferulic acid and studied isoferulates.

Concentration (mM)	Band (nm)	IFA	Mg(II) IFA	Mn(II)/Na(I) IFA
0.01	λ_max1_	322	316	315
	λ_max2_	294	291	290
	λ_max3_	243	239	235

**Table 5:** Antioxidant properties of isoferulic acid (IFA), Mg(II) IFA, and Mn(II)/Na(I) IFA determined using ABTS and CUPRAC assays (the concentration of tested substances in the samples was: * 10 μM, ** 20 μM).

Compound	ABTS * I%	ABTS ** I%	CUPRAC */C_Trolox_ [μM]	CUPRAC**/C_Trolox_ [μM]
IFA	65.96 ± 1.49	64.79 ± 1.37	40.92 ± 3.79	46.31 ± 5.90
Mg(II) IFA	65.83 ± 1.33	66.81 ± 2.24	87.93 ± 3.85	191.38 ± 8.30
Mn(II)/Na(I) IFA	51.95 ± 1.87	58.91 ± 1.51	105.85 ± 3.72	245.14 ± 2.41

**Table 6:** Cell viability inhibition in HaCaT cells treated with Mn(II)/Na(I) and Mg(II) complexes of IFA in the highest-obtained concentrations, versus the toxicity of IFA. VI = viability inhibition. The VI is measured versus the non-treated cell culture. All the values differ significantly from the control at the significance level at α = 0.05, as shown by the *t*-test.

Compound	Concentration (μM)	VI (%)	*t*-Test
IFA	1582	87 ± 5	0.010
Mg(II) IFA	16	87 ± 4	0.026
Mn(II)/Na(I) IFA	45	87 ± 5	0.009

## Data Availability

The data presented in this study are available on request from the corresponding author.

## Supplementary Materials

The following are available online at https://www.mdpi.com/article/10.3390/ma14123236/s1, Table S1: Selected bond distances (Å), bond angles (°), and torsion angles (°) in studied structures, Table S2: Geometries of hydrogen bonds and selected short contacts for Mg(II) and Mn(II)/Na(I) IFAs, Figure S1: FT-IR spectrum of: A. isoferulic acid, B. Mg(II) IFA, and C. Mn(II)/Na(I) IFA registered in the range of 400–4000 cm^−1^ for solid samples in the KBr matrix pellet, Figure S2: FT-Raman spectrum of: A. isoferulic acid, B. Mg(II) IFA, and C. Mn(II)/Na(I) IFA registered in the range of 400–4000 cm^−1^ for solid samples, Figure S3: The UV/VIS spectra of the isoferulic acid and its complexes with magnesium and manganese(II)/sodium(I) in methanol (0.01 mM).
