# Supplementary material for: Crystal Structure, Spectroscopic Characterization, Antioxidant and Cytotoxic Activity of New Mg(II) and Mn(II)/Na(I) Complexes of Isoferulic Acid
Source: Materials (Basel). 2021 Jun 11;14(12):3236. doi: 10.3390/ma14123236 (PMC8231219; doi:10.3390/ma14123236)
Supplement: Supplementary file 1 [file materials-14-03236-s001.zip › materials-1224289-SM.pdf]

Supplementary materials

Crystal structure, spectroscopic characterization, antioxidant and cytotoxic activity of new Mg(II) and Mn(II)/Na(I) complexes of isoferulic acid

Monika Kalinowska [1,*], Ewelina Gołębiewska [1], Liliana Mazur [2], Hanna Lewandowska [3], Marek Pruszyński [3,4], Grzegorz Świderski [1], Marta Wyrwas [1], Natalia Pawluczuk [1] and Włodzimierz Lewandowski [1]

[1] Department of Chemistry, Biology and Biotechnology, Institute of Civil Engineering and Energetics, Faculty of Civil Engineering and Environmental Science, Bialystok University of Technology, Wiejska 45E Street, 15-351 Bialystok, Poland; m.kalinowska@pb.edu.pl (M.K.); e.golebiewska@pb.edu.pl (E.G.); g.swiderski@pb.edu.pl (G.Ś.); w.lewandowski@pb.edu.pl (W.L.)

[2] Department of General and Coordination Chemistry and Crystallography, Institute of Chemical Sciences, Faculty of Chemistry, Maria Curie-Skłodowska University, M. C. Skłodowskiej Sq. 2, 20-031 Lublin, Poland (l.mazur@poczta.umcs.lublin.pl)

[3] Institute of Nuclear Chemistry and Technology, 16 Dorodna street, 03-195, Warsaw, Poland; h.lewandowska@ichtj.waw.pl (H.L.); m.pruszynski@ichtj.waw.pl (M.P.)

[4] NOMATEN Centre of Excellence, National Centre of Nuclear Research, 7 Andrzeja Soltana street, 05-400, Otwock, Poland

* Correspondence: m.kalinowska@pb.edu.pl

Table S1. Selected bond distances (Å), bond angles (°) and torsion angles (°) in studied structures.

**Mg(II) IFA**			
C1–O1	1.277(1)	Mg1–O1w	2.066(1)
C1–O2	1.271(1)	Mg1–O2w	2.098(1)
		Mg1–O3w	2.042(1)
O1w–Mg1–O3w	97.23(4)	O3w$^{(i)}$–Mg1–O1w	88.71(4)
O1w–Mg1–O2w	85.07(4)	O1w$^{(i)}$–Mg1–O1w	171.81(6)
O2w–Mg1–O3w	89.50(4)	O3w$^{(i)}$–Mg1–O2w	172.66(4)
O2w$^{(i)}$–Mg1–O2w	94.33(5)	O1w$^{(i)}$–Mg1–O2w	89.35(4)
O3w$^{(i)}$–Mg1–O3w	87.40(6)		
O1–C1–C2–C3	-176.8(1)	C2–C3–C4–C9	-174.9(1)
C1–C2–C3–C4	174.8(1)	C5–C4–O4–C10	-1.0(2)
**Mn(II)/Na(I) IFA**			
C1–O1	1.290(2)	C21–O9	1.263(2)
C1–O2	1.229(3)	C21–O10	1.270(2)
C11–O5	1.255(2)	C31–O13	1.262(3)
C11–O6	1.273(2)	C31–O14	1.268(2)
Mn1–O1	2.172(2)	Mn2–O4w$^{(ii)}$	2.151(2)
Mn1–O6	2.141(1)	Na1–O1$^{(iii)}$	2.376(2)
Mn1–O14	2.166(1)	Na1–O3	2.514(2)
Mn1–O1w	2.155(2)	Na1–O4	2.374(2)
Mn1–O2w	2.140(2)	Na1–O14$^{(iii)}$	2.394(2)
Mn1–O3w	2.288(2)	Na1–O15$^{(iv)}$	2.437(2)
Mn2–O10	2.162(1)	Na1–O16$^{(iv)}$	2.382(2)
Mn2–O3w	2.229(2)		
O2w–Mn1–O1w	85.36(6)	O10–Mn2–O10$^{(ii)}$	180.0
O6–Mn1–O1w	94.73(6)	O4w$^{(ii)}$–Mn2–O3w	88.82(6)
O2w–Mn1–O14	106.09(6)	O10$^{(ii)}$–Mn2–O3w	91.71(5)
O6–Mn1–O14	167.41(6)	O3w$^{(ii)}$–Mn2–O3w	180.0
O1w–Mn1–O14	89.08(6)	O4–Na1–O3	64.82(5)
O2w–Mn1–O1	91.97(6)	O4–Na1–O1$^{(iii)}$	102.22(6)
O6–Mn1–O1	95.50(5)	O4–Na1–O14$^{(iii)}$	174.40(6)
O1w–Mn1–O1	169.23(6)	O1$^{(iii)}$–Na1–O3	84.84(6)
O14–Mn1–O1	81.63(5)	O14$^{(iii)}$–Na1–O3	111.40(6)
O2w–Mn1–O3w	163.32(6)	O4–Na1–O16$^{(v)}$	85.16(6)
O5–Mn1–O3w	84.53(5)	O4–Na1–O15$^{(v)}$	100.68(6)
O1w–Mn1–O3w	81.62(6)	O16$^{(v)}$–Na1–O3	90.54(6)
O14–Mn1–O3w	84.16(5)	O15$^{(v)}$–Na1–O3	153.36(6)
O1–Mn1–O3w	102.69(6)	O1$^{(iii)}$–Na1–O16$^{(v)}$	168.55(7)
O4w–Mn2–O10	86.39(6)	O1$^{(iii)}$–Na1–O14$^{(iii)}$	72.94(5)
O4w–Mn2–O3w	91.18(6)	O1$^{(iii)}$ –Na1–O15$^{(v)}$	121.07(6)
O10–Mn2–O3w	88.29(5)	O14$^{(iii)}$–Na1–O15$^{(v)}$	84.41(6)
O4w–Mn2–O4w$^{(ii)}$	180.0	O16$^{(v)}$–Na1–O14$^{(iii)}$	99.20(6)
O4w–Mn2–O10$^{(ii)}$	93.61(6)	O16$^{(v)}$–Na1–O15$^{(v)}$	65.11(5)
O1–C1–C2–C3	-174.04(19)	C2–C3–C4–C9	3.9(4)
C1–C2–C3–C4	177.8(2)	C8–C7–O4–C10	12.0(3)
O6–C11–C12–C13	0.7(3)	C12–C13–C14–C19	-178.5(2)
C11–C12–C13–C14	-177.0(2)	C18–C17–O8–C20	-0.7(3)
O9–C21–C22–C23	-178.89(19)	C22–C23–C24–C29	174.0(2)
C21–C22–C23–C24	177.92(19)	C28–C27–O12–C30	-0.8(3)
O13–C31–C32–C33	-172.9(2)	C32–C33–C34–C39	-173.7(2)
C31–C32–C33–C34	-178.2(2)	C38–C37–O16–C40	3.4(3)

Symmetry codes: (i) -x, y, -z+1/2; (ii) -x+1, -y+1, -z+1; (iii) -x+1, -y, -z; (iv) x+1, y, z; (v) x+1, y, z-1

Table S2. Geometries of hydrogen bonds and selected short contacts for Mg(II) and Mn(II)/Na(I) IFAs.

Interaction	d_{D-H} /Å	$d_{H\cdots A}$ / Å	$d_{D\cdots A}$ / Å	<D-H···A /°	Symmetry code
			**1**		
O3–H3o···O4	0.79(2)	2.24(2)	2.665(1)	114(2)	
O3–H3o···O4w	0.79(2)	2.02(2)	2.771(1)	159(2)	-x+1/2, -y-1/2, -z+1
O1w–H1w···O1	0.85(2)	1.92(2)	2.766(1)	174(2)	
O1w–H1w···O6w	0.86(2)	1.95(2)	2.797(1)	170(2)	x, -y+1, z+1/2
O2w–H3w···O2	0.89(2)	1.78(2)	2.662(1)	171(2)	
O2w–H4w···O5w	0.84(2)	1.91(2)	2.746 (1)	174(2)	x, y-1, z
O3w–H5w···O6w	0.86(2)	1.88(2)	2.730(1)	171(2)	
O3w–H6w···O5w	0.82(2)	1.92(2)	2.740(1)	174(2)	
O4w–H7w···O1	0.89(2)	1.81(2)	2.685(2)	168(2)	
O4w–H8w···O2	0.80(2)	2.07(2)	2.845(1)	162(2)	x, -y, z+1/2
O5w–H9w···O2w	0.85(2)	2.15(2)	2.948(1)	158(1)	-x, -y+1, -z
O5w–H10w···O1	0.94(2)	1.88(2)	2.785(1)	164(2)	-x, y+1, -z+1/2
O6w–H12w···O2	0.86(2)	2.00(2)	2.835(1)	163(2)	x, y+1, z
O6w–H11w···O4w	0.86(2)	1.89(2)	2.734(1)	167(2)	x, -y, z-1/2
C8–H8···O3	0.96(1)	2.59(1)	3.399(2)	143(1)	x, -y, z-1/2
			**2**		
O1w–H1w···O11	0.90(4)	1.85(4)	2.741(2)	171(3)	x, y, z+1
O1w–H2w···O10	0.78(3)	2.02(3)	2.793(2)	171(3)	-x+1, -y+1, -z+1
O2w–H3w···O1	0.80(3)	2.01(3)	2.807(2)	174(3)	-x+1, -y, -z+1
O2w-H4w···O5	0.85(3)	1.90(3)	2.699(2)	157(3)	
O3w–H5w···O9	0.87(3)	1.73(3)	2.581(2)	168(3)	
O3w–H6w···O13	0.88(3)	1.76(3)	2.619(2)	167(3)	
O4w–H7w···O7	0.80(3)	1.99(3)	2.777(2)	168(3)	x-1, y, z
O4w–H8w···O6	0.79(4)	1.92(4)	2.694(2)	169(4)	-x+1, -y+1, -z+1
O3–H3o···O9	0.80(3)	1.95(3)	2.745(2)	171(3)	-x+1, -y, -z
O7–H7o···O13	0.79(3)	1.84(3)	2.607(2)	167(3)	x+1, y, z
O11–H11o···O2	0.88(3)	1.76(3)	2.639(2)	176(3)	-x+1, -y, -z
O15–H15o···O5	0.83(3)	1.83(3)	2.652(2)	171(3)	x-1, y, z
C5–H5···O9	0.93	2.63	3.253(3)	125	-x+1, -y, -z
C25–H25···O2	0.93	2.66	3.309(3)	128	-x+1, -y, -z
C40–H40c···O3	0.96(2)	2.60(3)	3.418(3)	144(2)	x-1, y, z+1

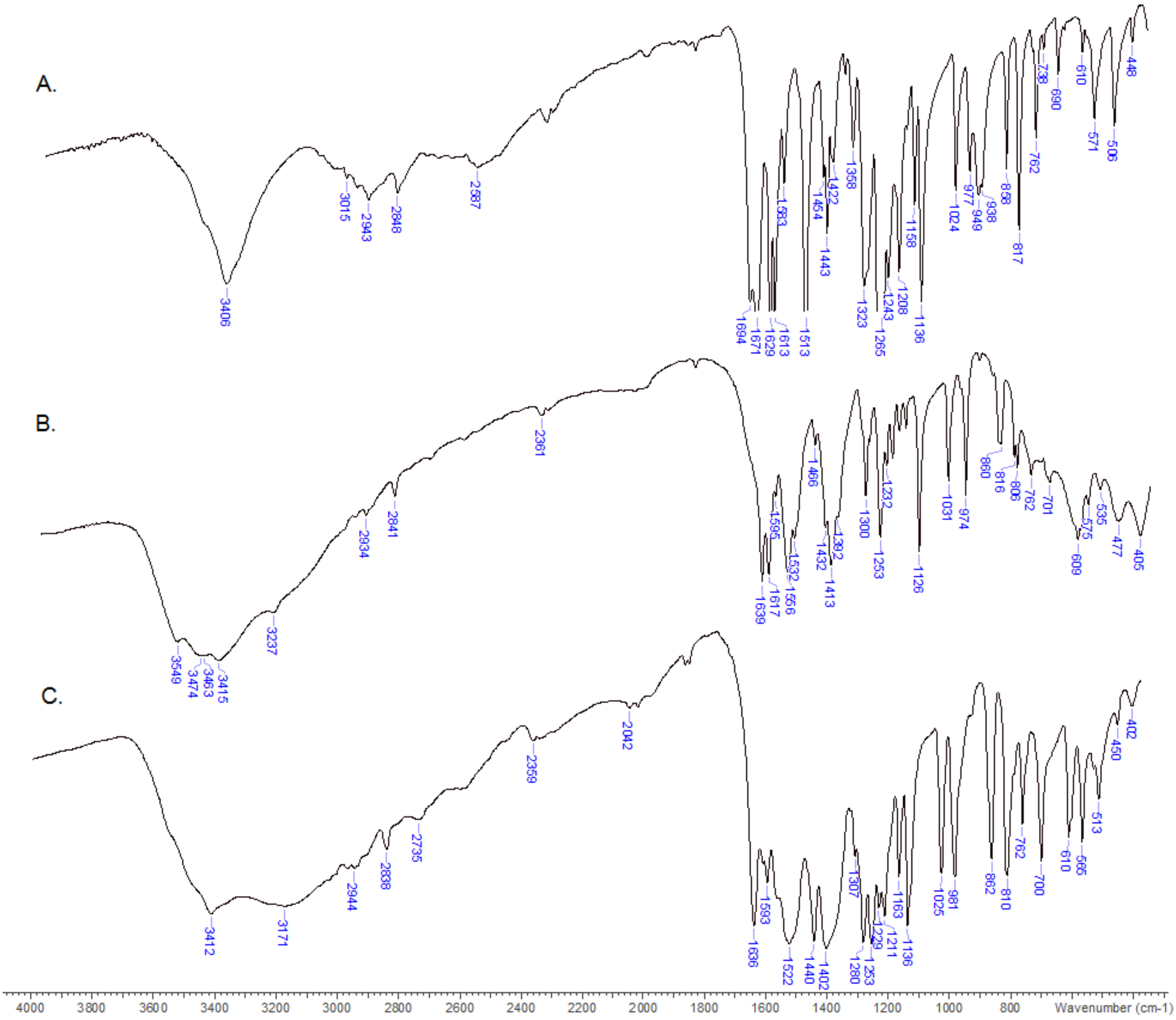

Figure S1. FT-IR spectrum of: A. isoferulic acid, B. Mg(II) IFA and C. Mn(II)/Na(I) IFA registered in the range of 400-4000 cm^{-1} for solid samples in the KBr matrix pellet.

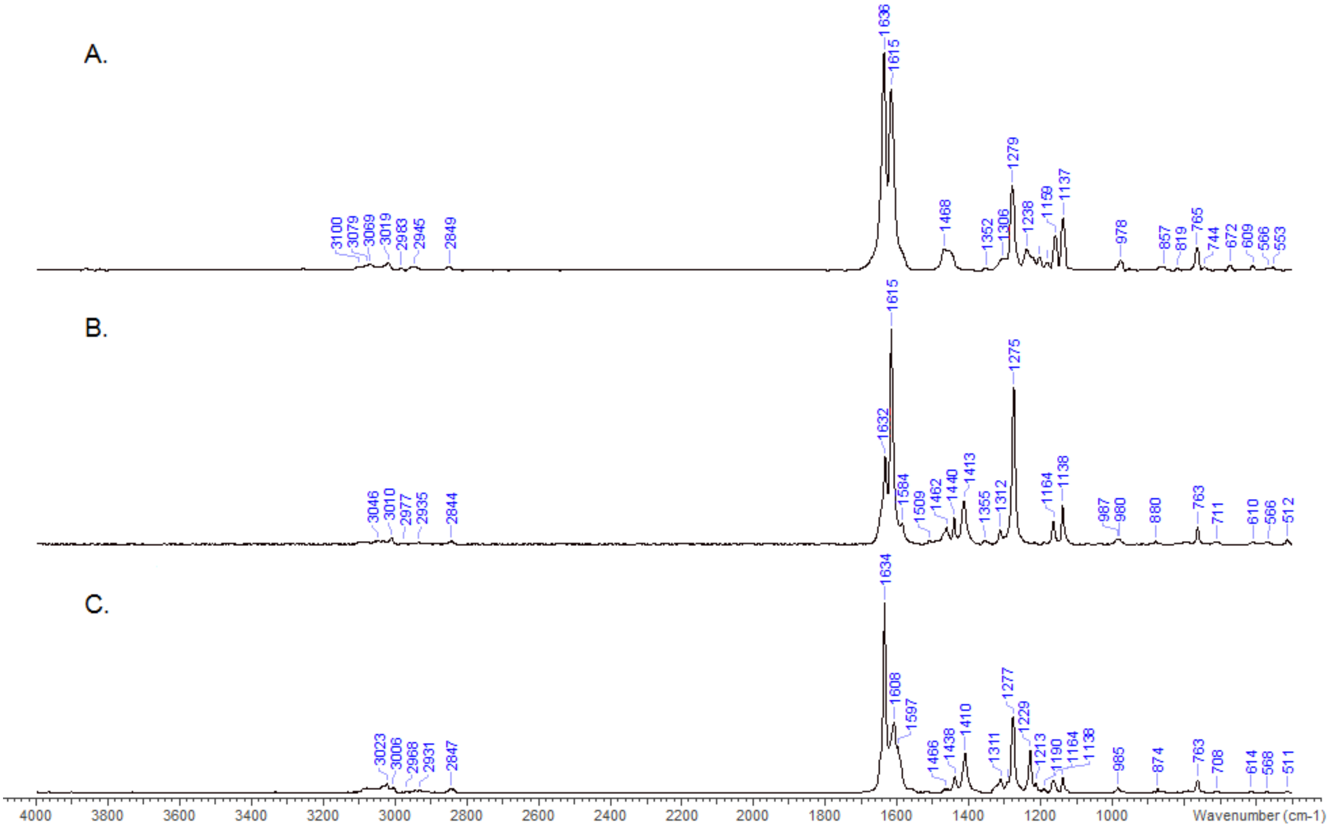

Figure S2. FT-Raman spectrum of: A. isoferulic acid, B. Mg(II) IFA and C. Mn(II)/Na(I) IFA registered in the range of 400-4000 cm^{-1} for solid samples.

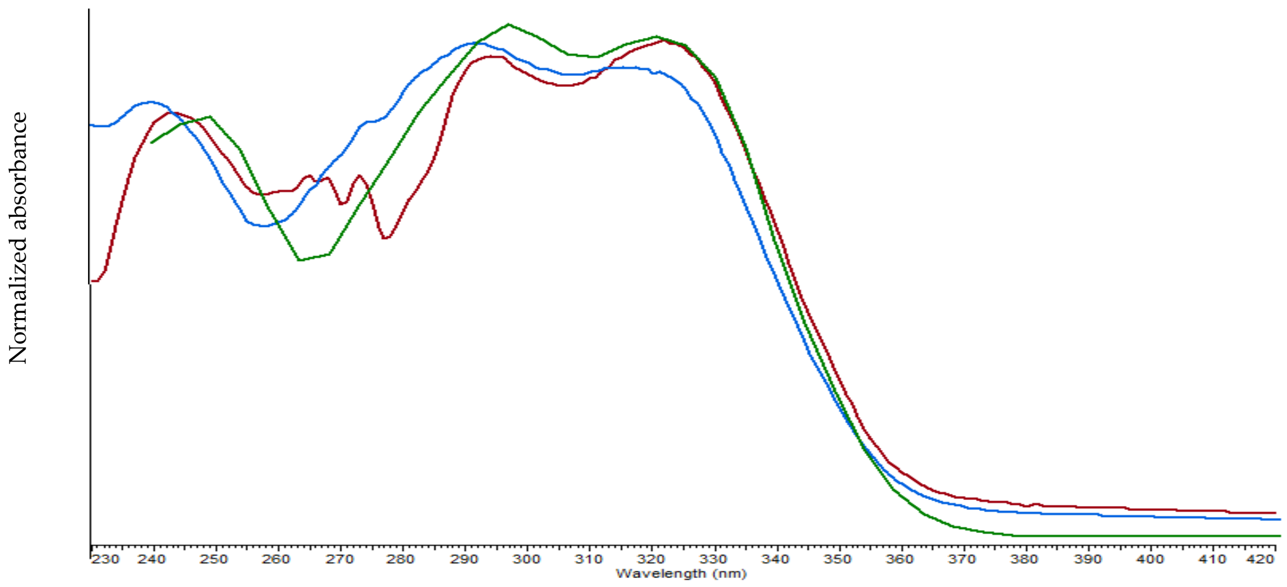

Figure S3. The UV/VIS spectra of the isoferulic acid (red line) and it complexes with magnesium (green line) and manganese(II)/sodium(I) (blue line) in methanol (0.01 mM).
